# Deformation of Aflibercept and Ranibizumab Syringes Causes Variation in Intravitreal Injection Volume and Risks Retinal Tear Formation

**DOI:** 10.1016/j.xops.2022.100202

**Published:** 2022-07-15

**Authors:** Joseph J. Raevis, Colin A. Lemire, David J. Ramsey, James Riccobono, Efren Gonzalez

**Affiliations:** 1 Department of Ophthalmology, Beth Israel Deaconess Medical Center, Boston, Massachusetts; 2 Department of Ophthalmology, Harvard Medical School, Boston, Massachusetts; 3 Division of Ophthalmology, Department of Surgery, Lahey Hospital & Medical Center, Peabody, Massachusetts; 4 Department of Ophthalmology, Tufts University School of Medicine, Boston, Massachusetts

**Keywords:** Intravitreal injection, Syringe, Variability, Volume, aflibercept, CI, confidence interval, PFS, prefilled syringe, TB, tuberculin

## Abstract

**Purpose:**

The intravitreal injection volume is known to vary with plunger alignment and the speed of injection. We investigated the role that syringe stopper deformation plays in allowing excess volumes to be injected into the eye and the potential for the vitreous humor to become incarcerated when excess force is released within the eye.

**Design:**

Experimental study.

**Methods:**

Aflibercept prefilled syringes (PFSs), ranibizumab PFSs, and 1-ml tuberculin (TB) syringes were subjected to increasing injection force to assess the extent to which each design allowed for excess volumes to be expelled after the stopper reached the bottom of the syringe barrel (i.e., after the 50-μl dose was expelled).

**Main Outcome Measures:**

Additional volume expelled with stopper deformation.

**Results:**

Syringe stoppers are capable of deformation into the dead space when additional force is applied. This allows for progressively greater medication doses to be administered. At an additional force of 3.92 N after the syringe stopper came in contact with the bottom of the syringe barrel, the aflibercept PFSs, ranibizumab PFSs, and 1-ml TB syringes dispensed an additional 17.2%, 11.4%, and 0.8% higher volume than the intended volume of 50 μl, respectively. Upon release of this force, a proportional volume was observed to be drawn back into the needle.

**Conclusions:**

The intravitreal injection volume varies with the force applied to fully depressed syringes because of syringe stopper deformation. We advise that performing forceful intravitreal injections be avoided to prevent excessive dosing of medication. We also caution that pressure applied to the plunger during intravitreal injections not be released while the needle is in the vitreous cavity to guard against vitreous incarceration, which could lead to retinal tear formation or detachment.

Agents targeting VEGF have been approved for the intravitreal administration of 50 μl of medications; however, studies have shown that in practice, there is significant variation in the actual volumes delivered.^1^, ^2^, ^3^, ^4^ Several factors have been proposed to account for the risk of excess volume administration, including the large internal diameter of prefilled syringes (PFSs), which magnifies errors related to misalignment of the plunger with the relatively thick mark designed to set the dose^1^^,^^3^ and, more recently, the speed at which intravitreal injections are administered.^3^ This lack of precision is concerning because it poses the risks of inaccurate dosing and side effects such as elevated intraocular pressure, which, in severe cases, can induce transient vision loss, especially with aflibercept PFSs.^1^^,^^3^

In this report, we identified an additional controllable factor impinging on the accuracy and safety of intravitreal injection of aflibercept and ranibizumab specific to their delivery by their respective PFSs. We showed that the stoppers in these devices easily deform with even modest amounts of force applied after they have reached their intended end points. This may lead to the inadvertent administration of additional medication or, potentially even more concerning, reflux of material into the syringe when this force is released. In contrast, standard 1-ml tuberculin (TB) syringes are often used for the administration of bevacizumab and, in some practices, for the administration of aflibercept and ranibizumab before packaging of these medications in PFSs. On the basis of our observations, we recommend that excess force not be applied to PFSs at the time of intravitreal injections and, more importantly, that any force applied to these syringes not be released while the needle is within the vitreous cavity to guard against incarceration of the vitreous humor from negative pressure brought about by the elastic rebound of the PFS stoppers.

## Methods

Three types of syringes were examined in this study: (1) polycarbonate glass PFSs designed for the administration of aflibercept (0.3 mg of Eylea; Regeneron Pharmaceuticals); (2) polycarbonate glass PFSs designed for the administration of ranibizumab (0.3 mg of Lucentis; Genentech); and (3) polypropylene plastic syringes containing 1 ml of TB, with a Luer-lock tip (Monoject; Cardinal Health Inc) that held bevacizumab (Avastin; Genentech) prepared by the Beth Israel Deaconess Medical Center compounding pharmacy. All the syringes were flushed with deionized water before evaluation. This study did not involve human subjects, and therefore, we did not seek institutional review board review.

To measure the force required to fully depress each syringe type using a 32-gauge needle attached, each unit was backfilled with deionized water, held vertically in a brace, and 50 g of weight (0.49 N) was sequentially added to the plunger rod until the stopper started slowly moving toward to distal aspect of the syringe, causing the 50-μl dose to be expelled. After the syringe stopper reached the distal aspect of the syringe barrel, we waited for an additional 30 seconds to ensure that equilibrium was reached and no further fluid was detected. This step ensured that all 3 syringe types had their stoppers fully depressed using the minimum amount of force. Next, additional weights were applied in 50-g increments to achieve a total additional force of 3.92 N beyond the minimum force required to achieve a fully depressed plunger. The deionized water that was expelled from the syringe after each additional 50-g weight was added for 30 seconds was weighed on an MS104S balance (Mettler-Toledo Inc), with a precision of 0.1 mg. The volume dispensed from the syringe was calculated on the basis of the known density of water (1 g/ml). A total of 36 trials were performed, 12 for each syringe type, with half of these performed at 4° C and half at room temperature (20°C to 22°C).

To understand the mechanism behind the tendency of each syringe to dispense additional fluid, each type of syringe was sectioned to permit direct visualization of the plunger unimpeded by the refraction of light by the curved syringe wall. The Crescent Nicholson hand file (Apex Tool Group, LLC) was used to remove 45% of the distal aspect of each type of PFS, and the X-ACTO blade (Elmer’s Products, Inc) was used to remove a similar amount of the distal aspects of the 1-ml TB syringe. The stopper of each syringe was then fully depressed to the distal aspect of the barrel using the requisite amounts of weight previously determined. As approximately 3.92 N of additional weight was gradually applied, the compression of the stopper was then recorded by means of videography and photography using the Galaxy A52 device (Samsung USA). The images obtained were aligned using i2k-Retina (version 3.0.0.107; Dual-Align LLC) and the difference in the distal position of the stopper between the 2 images highlighted by overlaying the 2 images using Photoshop (version 21.1.2, Adobe Systems).

### Statistics

Data were recorded using Microsoft Excel 2010 (version 14.0, Microsoft Corporation) and analyzed using RStudio, version 1.1.422 (RStudio: Integrated Development for R. RStudio, PBC). The Student *t* test was used to perform pairwise comparisons between the means, whereas the analysis of variance was used to compare the means among the 3 types of syringes. The Wilcoxon signed rank test was used to compare the results obtained at different temperatures. All the tests were 2-sided, and *P* values of < 0.05 were regarded as statistically significant.

## Results

The force required to initiate depression in each syringe type using a 32-gauge needle attached was approximately 0.5, 1.0, and 3.0 N for the ranibizumab PFS, aflibercept PFS, and TB syringe, respectively.

**Figure 1:**
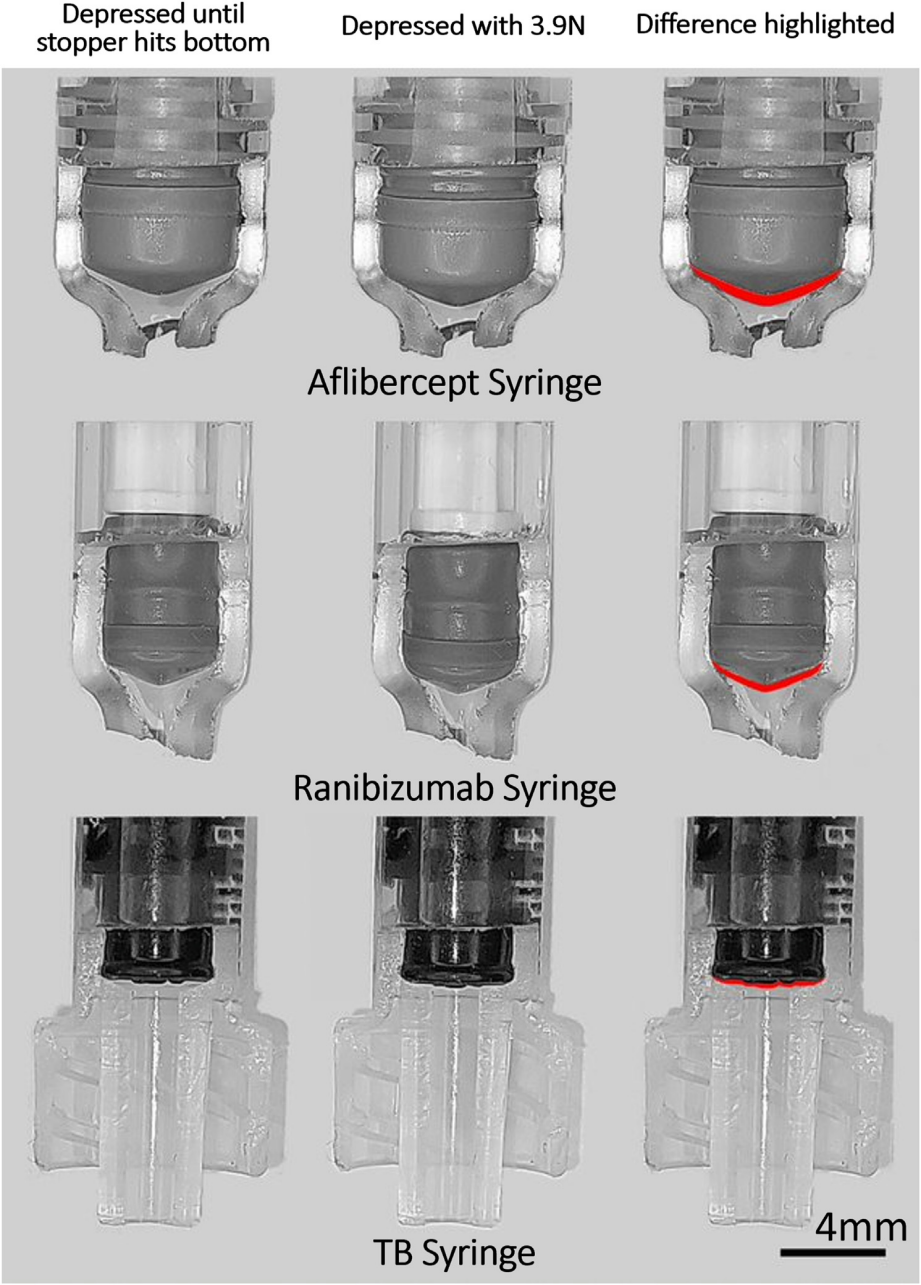
Qualitative analysis of an aflibercept prefilled syringe, a ranibizumab prefilled syringe, and a 1-ml tuberculin (TB) syringe with approximately half of the syringe wall removed to facilitate viewing of the syringe stoppers. The syringes were depressed until the stopper reached the bottom of the syringe barrel (left column), after which an additional force of 3.9 N was applied (middle column). The difference between these 2 states is highlighted in red (right column), illustrating the change in shape of the syringe stoppers.

**Figure 2:**
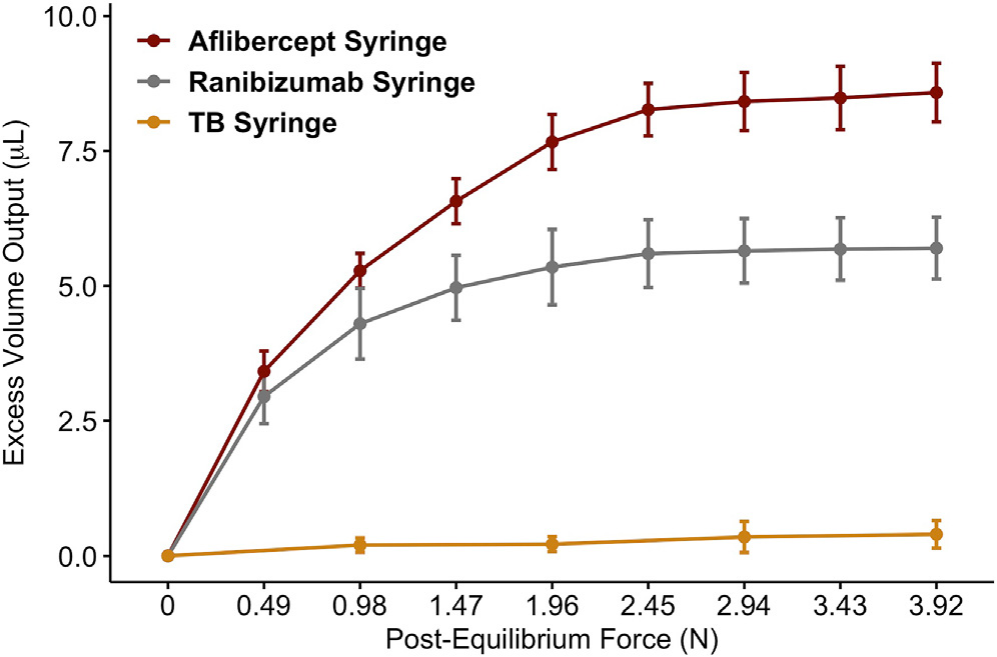
Additional volume dispensed from an aflibercept prefilled syringe, a ranibizumab prefilled syringe, and a 1-ml tuberculin (TB) syringe as increments of 0.49 N (50 g) or 0.98 N (100 g) of force were applied. This force was applied in addition to the amount of force required to fully depress the syringe. Paired *t* tests were used to identify significant differences between each syringe pair at all time points examined (*P* < 0.001).

Next, with each syringe stopper already fully depressed to the distal aspect of the barrel (i.e., with the 50-μL dose expelled), we assessed the propensity of the stopper to deform as additional weight was added (Fig 1; Video 1, available at www.opthalmologyscience.org). With a force of 0.98 N (100 g) applied, the aflibercept PFS expelled an additional 5.28 μl (95% confidence interval [CI], 4.96–5.60 μl [10.57%; 95% CI, 9.92%–11.21%]), the ranibizumab PFS expelled an additional 4.30 μl (95% CI, 3.65–4.95 μl [8.60%; 95% CI, 7.29%–9.91%]), and the 1-ml TB syringe expelled an additional 0.20 μl (95% CI, 0.07–0.33 μl [0.40%; 95% CI, 0.13%–0.67%]) of additional volume beyond the 50-μl target (Fig 2). With an additional force of 3.92 N (400 g) applied, an additional 8.58 μl (95% CI, 8.04–9.13 μl [17.17%; 95% CI, 16.08%–18.26%]), 5.70 μl (95% CI, 5.13–6.27 μl [11.40%; 95% CI, 10.26%–12.54%]), and 0.40 μl (95% CI, 0.14–0.66 μl [0.80%; 95% CI, 0.29%–1.31%]) of excess volume was expelled from the aflibercept PFS, ranibizumab PFS, and 1-ml TB syringe, respectively. One-way analysis of variance revealed that the mean total excess output was significantly different among the 3 syringe types (F = 495.6, *P* < 0.001). When this experiment was repeated at 4**°** C, no difference was found in the amount of additional fluid expelled (aflibercept PFS, *P* = 0.81; ranibizumab PFS, *P* = 0.39; and 1-ml TB syringe, *P* = 1.00).

**Figure 3:**
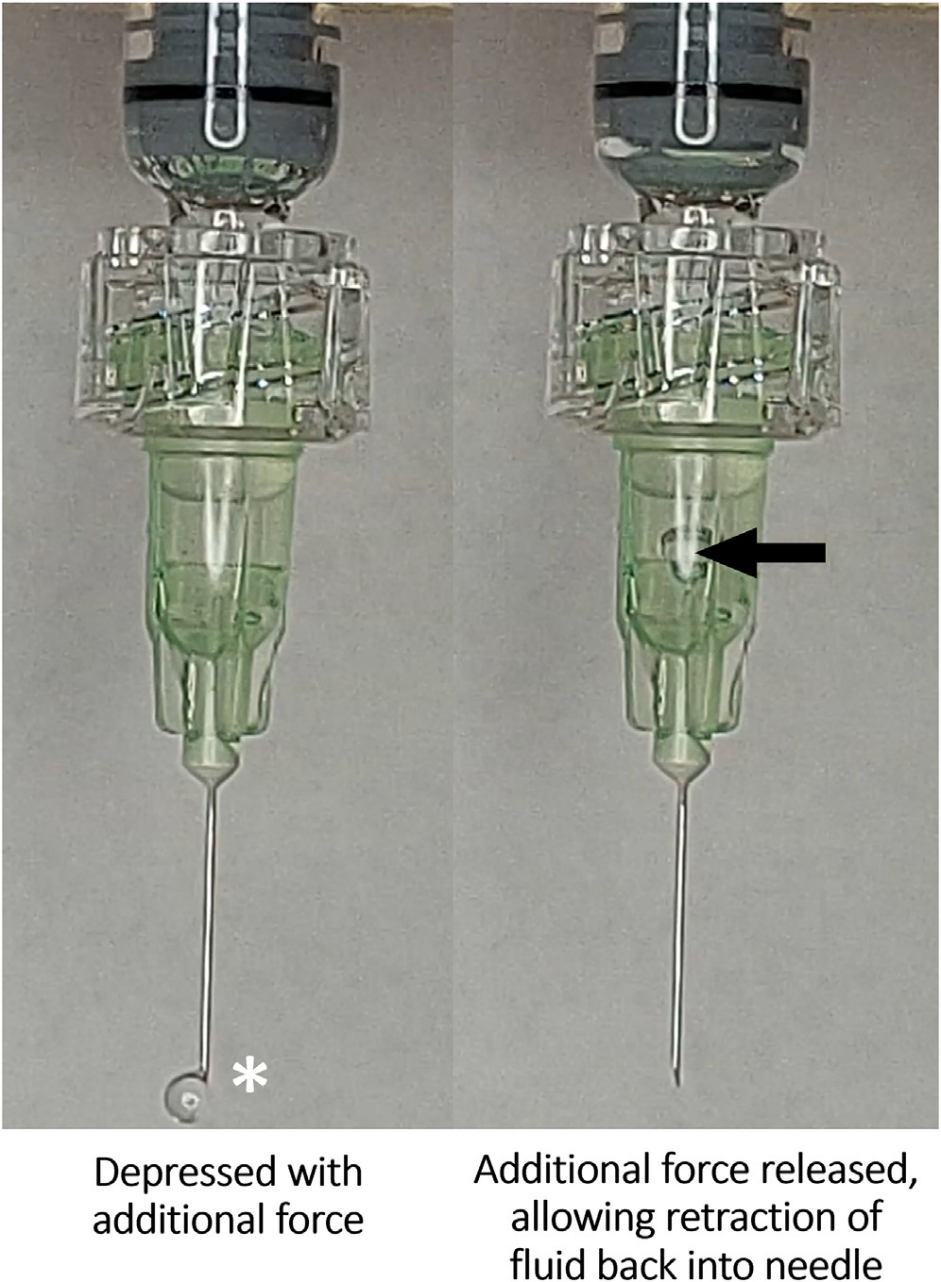
**A,** When additional force is applied to a fully depressed syringe with the stopper resting at the bottom of the syringe barrel, more fluid is capable of being expelled (∗). **B,** When the additional force is released, the syringe stopper rebounds to its original position, allowing retraction of the fluid at the tip of the needle along with a small amount of air (black arrow).

Finally, when the additional force applied was suddenly released, the elastic recoil generated a negative pressure sufficient to draw fluid back into the syringe barrel (Fig 3; Video 2, available at www.opthalmologyscience.org). If this recoil is allowed to occur outside the eye, an air bubble is often visible. In clinics, this phenomenon is often observed as an air bubble in the syringe when excess pressure applied during an intravitreal injection is released outside the eye.

## Discussion

In this study, we explored the role that deformation of syringe stoppers plays in metering the volume of medication injected. We compared the aflibercept PFS, ranibizumab PFS, and 1-ml TB syringe, which is commonly used to administer bevacizumab. This work was inspired by the observation that additional fluid can often be expelled after intravitreal injections by applying additional force to the syringe outside the eye. This study demonstrated that additional force applied deforms the syringe stopper at the end of the plunger and can result in a significant, unintentional amount of additional medication to be administered during intravitreal injections. This ranged from as much as 17% for the aflibercept PFS to as little as < 1% for the 1-ml TB syringe above the intended 50-μl volume when an additional force of 3.92 N (400 g) was applied to the syringe after the plunger reached the end of the syringe barrel. We chose an additional force of approximately 4 N to apply to the syringe plungers because Lee et al^3^ showed that to depress aflibercept, ranibizumab, and TB syringe plungers fully at a speed of 2.66 mm/second, a force of approximately 3 to 6 N was required. An additional force of 4 N is easy to imagine for a physician to apply during an intravitreal injection.

Four factors may account for this phenomenon and explain the differences between the syringe types:

1. The internal diameter and area of the aflibercept PFS barrel (6.26 mm and 30.8 mm^2^, respectively) are significantly larger than those of the ranibizumab PFS barrel (4.63 mm and 16.8 mm^2^, respectively) and 1-ml TB syringe barrel (4.76 mm and 17.8 mm^2^, respectively).^3^ A wider opening allows more room for stopper deformation into the distal aspect or dead space of the syringe.
2. Both aflibercept and ranibizumab PFSs have tapered, dome-shaped stoppers at the distal end of their syringe barrels, whereas both the stopper and the distal end of 1-ml TB syringes are nearly flat. This likely contributes to the “hard” stop one experiences while performing injections using 1-ml TB syringes, making it easier for physicians to know when to stop applying force to the syringe.
3. The aflibercept and ranibizumab PFS stoppers are hollow. In comparison, the 1-ml TB syringe stopper is solid, thereby limiting its propensity to deform.
4. The aflibercept and ranibizumab PFS stoppers are made of a material different from the 1-ml TB syringe stopper.

### Side Effects of Excess Volume

It has been shown that patients receiving > 7 intravitreal injections annually are at a higher risk of needing glaucoma surgery.^5^ Excess intravitreal volumes may have adverse effects, such as excessive intraocular pressure, leading to transient vision loss,^1^^,^^3^ and those with advanced glaucoma or a low ocular perfusion pressure may be more susceptible to such complications.^1^

### Potential Mechanism for Retinal Tear Formation

Storey et al^6^ showed that there is approximately a 1:7500 chance of a retinal tear developing after an intravitreal injection of an anti-VEGF agent. However, no association was found between the various medications examined and the rate of retinal tear formation. The rarity of retinal tears and the many factors that contribute to their formation may account for this finding.

**Figure 4:**
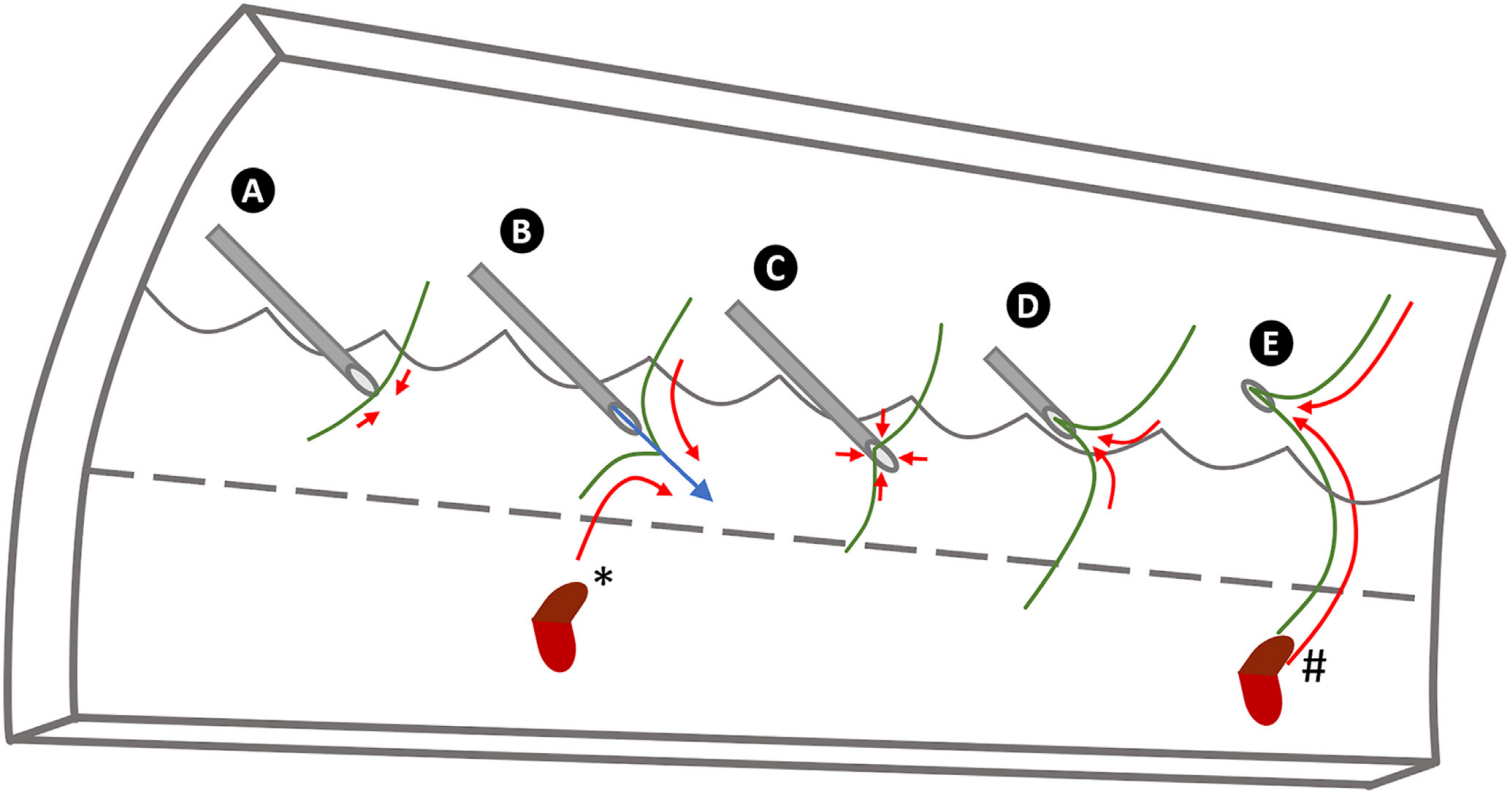
Proposed mechanisms for intravitreal injections to cause vitreoretinal traction capable of inducing a retinal tear. During an intravitreal injection, a retinal tear (∗) may be created when the needle is passed through the pars plana (**A**) or at the time of medication injection (**B**). An alternative hypothesis, one supported by the present study, is that the vitreous humor may be drawn into the needle because of the negative pressure created by the recoil of the syringe stopper after medication administration (**C**). A retinal tear (#) forms as the needle is withdrawn from the eye because of vitreoretinal traction (**D**, **E**).

When additional force applied to a plunger is released, the stopper retracts to its original position, creating vacuum, which causes the fluid to retract into the needle and syringe. As fluid is drawn back into the needle, the vitreous humor could become incarcerated, and withdrawal of the needle from the eye could lead to the formation of a retinal tear (Fig 4). This proposed mechanism warrants further investigation. Nevertheless, we advise ophthalmologists performing intravitreal injections to consider not releasing pressure applied to the plunger while the needle is within the vitreous cavity to guard against potential vitreous incarceration via this mechanism.

### Ideal Syringe Design

**Figure 5:**
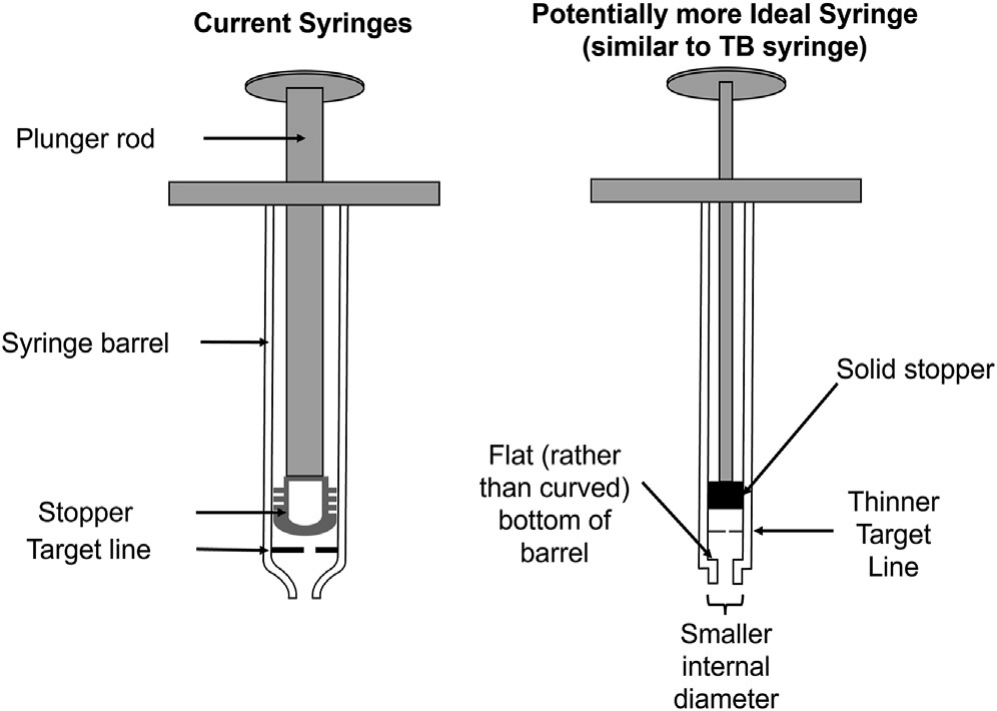
Model of a more ideal syringe combining design elements similar to that of a 1-ml tuberculin (TB) syringe with those of a prefilled syringe. A smaller internal diameter could improve the accuracy of dosing because any alignment errors between the target line on the syringe barrel or stopper would be minimized by the longer distance. A flat-bottom syringe barrel would lead to a “hard” stop, providing a tactile guide to know when to stop pushing. Both a flat bottom and solid stopper prone to less deformation would reduce the risk that an additional volume would be dispensed in the case of excess force application.

We propose the following design elements for a more “ideal” syringe for intravitreal injections (Fig 5):

1. Employ a thin, dashed line to improve the ease and accuracy of aligning the stopper with the 0.05-ml dose mark. Consider adding an additional mark on the syringe stopper to aid in this alignment.
2. Use a small(er) internal diameter to help mitigate the impact of physician errors in alignment with the dose mark. Gallagher et al^1^ showed that alignment errors result in an approximately twofold greater volume error when the 1-ml TB syringe was compared with the aflibercept PFS.
3. Use a stopper material that resists deformation (with a higher durometer quotient).
4. Use a stopper that is solid to prevent deformation.
5. Flatten the distal aspect of the syringe barrel to facilitate a “hard” stop. Having a flat-bottom syringe would also allow for easier syringe stopper alignment with the dose mark on the barrel compared with a tapered stopper.

### Study Limitations

To allow for direct visualization of the stopper, we had to remove a portion of the syringe wall from each syringe type. This may have accentuated bulging of the stopper into the available dead space. However, all quantitative studies were performed using fully intact syringes. Our studies of force applied were limited to increments of 0.49 N. A continuous force meter could have yielded more precise results; however, it was not available. Furthermore, it would have been unlikely to alter the conclusions of our study. The requirement of having to close the door on the balance to accurately obtain each measurement introduced a delay of approximately 30 seconds between each weight being applied and the fluid being weighed, which might have allowed for a small amount of evaporation. Other studies have shown that the speed of injection affects the amount of force needed to depress the syringe.^3^ Our experimental design limits our ability to assess this additional dimension. Finally, our tests were performed using deionized water rather than actual pharmacologic agents because of their cost. We cannot rule out the fact that differences in the viscosity or other properties of proprietary pharmaceutical preparations could alter some of the results obtained from our in vitro studies. With newer and more viscous drugs coming to market, future studies to address this concern will have added significance.

### Summary

The goal of intravitreal injections is to administer as safely and precisely as possible 50 μl of medication; however, multiple studies have shown that there is significant variation in the actual doses administered.^1^, ^2^, ^3^, ^4^ This study highlights the likely role of syringe stoppers and their deformation as a cause of dose variability and the risk of vitreous incarceration. This mechanism may also help explain many of the well-known side effects of intravitreal injections related to increases in intraocular pressure and retinal tear formation that can lead to retinal detachment.

## Supplementary Data

Video 1 VidClip of an aflibercept PFS, ranibizumab PFS, and 1 mL TB syringe each being manually depressed. When additional force is applied after the stopper reaches the end of the syringe barrel there is deformation of the stopper to varying degrees across the syringe types. Also note, when the force is released, the syringe stopper retracts.

Video 2 VidClip of when additional force is applied to a fully depressed syringe with the stopper resting at the bottom of the syringe barrel additional fluid is often capable of being expressed. More significantly, when this additional force is released, the syringe stopper rebounds to its original position, allowing retraction of the fluid at the tip of the needle to be retracted along with a small amount of air.
